# Development of an innovative methodology combining chemical fractionation and *in vivo* analysis to investigate the biological properties of cheese

**DOI:** 10.1371/journal.pone.0242370

**Published:** 2020-11-19

**Authors:** Guillaume Cardin, Isabelle Ripoche, Cyril Poupet, Muriel Bonnet, Philippe Veisseire, Pierre Chalard, Anne Chauder, Etienne Saunier, Julien Priam, Stéphanie Bornes, Laurent Rios

**Affiliations:** 1 Université Clermont Auvergne, INRAE, VetAgro Sup, UMRF, Aurillac, France; 2 Université Clermont Auvergne, CNRS, Sigma Clermont, ICCF, Clermont-Ferrand, France; 3 Dômes Pharma, Pont-du-Château, France; Institute for Biological Research "S. Stanković", University of Belgrade, SERBIA

## Abstract

With the ever-increasing human lifespan, age-related affections have become a public health issue. The health sector is looking for new bioactive compounds to respond to this demand. The unexplored microbial biodiversity and its metabolites represent a major source of innovative bioactive molecules with health potential. Fermented foods, such as raw-milk cheese, have already been investigated for their rich microbial environment, especially for their organoleptic qualities. But studies remain limited regarding their effects on health and few metabolites of microbial origin have been identified. An efficient methodology was developed in this study to investigate the biological effect of raw-milk cheese, combining a chemical fractionation, to isolate the most metabolites from the cheese matrix, and an *in vivo* biological test using *Caenorhabditis elegans*. *C*. *elegans* was brought into contact with cheese extracts, obtained by means of chemical fractionation, and with freeze-dried whole cheese by supplementing the nematode growth medium. A longevity assay was performed to evaluate the effects of the extracts on the worms. Our results demonstrate the feasibility of the method developed to bring the worms into contact of the cheese extracts. The evaluation of the effects of the extracts on the longevity was possible. Some extracts showed a beneficial effect as extract W70 for example, obtained with water, which increases the mean lifespan by 16% and extends the longevity by 73% (*p* < 0.0001).

## Introduction

Between 1981 and 2010, more than 50% of new drugs were derived from natural products [1]. However, to date, less than 15% of plant biodiversity [2] and less than 1% of the microbial biodiversity [3] have been explored. Thus, microorganisms represent a major source of innovative bioactive molecules with health potential. Some microorganisms have already been investigated for their biological properties, as probiotics for example [4, 5], and could be of great interest for preventive health applications. Natural compounds, especially from plants, are already being used for age-related affections (for examples cardiovascular diseases [6] or osteoarthritis [7]), that have become a public health issue with the increase in life expectancy. Many biological processes are involved in these affections including inflammation [8] and oxidative stress [9]. Some studies have demonstrated the anti-inflammatory [10] or antioxidative activities [11] of certain specific microorganisms, or of some of their metabolites. The unexplored microbial diversity may be a new source of bioactive compounds, with a potential beneficial effect against these age-related processes. Fermented foods, such as cheese, have been investigated for their rich microbial environment. This daily food, appreciated in many countries for its taste value, has been the subject of many nutritional [12, 13] and sensorial [14, 15] studies in an attempt to understand and to improve its organoleptic qualities. Recently, certain biological properties, such as the antimicrobial [16], probiotic [17, 18] and anti-inflammatory [19] activities of the bacteria isolated from milk, yogurt or cheese have been studied. However, these studies remain limited and few bioactive metabolites of microbial origin have been identified.

The goal of our study was to develop a methodology to isolate the metabolites from raw-milk cheese by obtaining cheese fractions, before evaluating their biological effect. Two extraction processes were employed to develop an efficient extraction of cheese metabolites. The first one consisted in a fractionation extraction based on the variation of the solvent polarity to remove the most compounds from the cheese matrix. The second process consisted in aqueous extractions, as it has been reported that the polar compounds in cheese possess potent biological properties [20, 21]. Moreover, water is considered a green solvent, making the process more environmentally suitable for industrial development if it presents good results. Then the biological effects of the resulting fractions were evaluated using the nematode *Caenorhabditis elegans* (*C*. *elegans*) as an *in vivo* model. The similarities in *C*. *elegans* and human genetics, as well as its short lifespan, make it a reliable experimental model to test the impact of such cheese extracts on the host [22]. Like *Drosophila melanogaster* this worm has become a rudimentary *in vivo* model used to characterise the probiotic properties of bacteria and yeasts [23–25] but its use has now been extended to the screening of the biological effects of plant extracts [26, 27]. In this context, we decided to use *C*. *elegans* to investigate the impact of cheese fractions on the longevity. A method was developed to bring the worms into contact with cheese extracts, as well as with freeze-dried whole cheese, by supplementing the usual nematode growth medium. Then, longevity assays using *C*. *elegans* were performed to evaluate the biological effects of the cheese extracts and of the whole cheese.

## Material and methods

### Cheese sample

Goat cheese, ripened for twenty days, (the simplest form made by allowing raw milk to naturally curdle and then draining and pressing the curds) was purchased from a local producer (Chèvrerie des Oliviers, Saint-Georges sur Allier, France), cut into small slices and stored at—25°C. Before extraction, the cheese was freeze-dried (FreeZone Triad Freeze Dryer, Labconco corporation, Kansas City, Missouri) and ground with mortar and pestle. The freeze-dried cheese (FDC) was stored at 4°C in a waterproof container.

### Reagents and solvents

Cyclohexane and dichloromethane were purchased from Carlo Erba (Val de Reuil, France), ethyl acetate and absolute ethanol were purchased from VWR chemicals (Radnor, USA). 5-fluoro-2’-deoxyuridine (FUdR), amphotericin B (250 μg/mL), agarose, cholesterol, NaCl, MgSO_4_, CaCl_2,_ Na_2_HPO_4_, KH_2_PO_4_, potassium phosphate buffer and NaOH were purchased from Sigma Aldrich (Saint-Louis, USA). Lysogeny Broth (LB, Miller’s Modification), peptone and agar were purchased from Conda (Madrid, Spain). Yeast extract was purchased from Fisher Scientific (Hampton, USA).

### Cheese extractions: Obtaining cheese fractions

#### Cryogenic crushing of freeze-dried cheese

The freeze-dried cheese (FDC) was crushed using cryogenic grinding. The resulting powder was kept in a waterproof container at -25°C until use.

A method was developed to extract efficiently metabolites from freeze-dried cheese, based on successive solid/liquid extractions. According to their polarity property, the solvents used were adapted for each extraction performed to remove the metabolites from the cheese matrix. Five different solvents were used to collect cheese metabolites as specified by a polarity scale, from nonpolar to polar (cyclohexane, dichloromethane, ethyl acetate, absolute ethanol and water). To make sure the cheese matrix was exhausted, each solvent extraction was performed three times successively (called separation step 1, 2 or 3 in this study). The yield of each solvent extraction and the separation step was determined. These values were used to quantify the amount of dry extract recovered and to check the efficiency of the exhaustion, by comparing the yield of each separation step for the same solvent.

**Fig 1:**
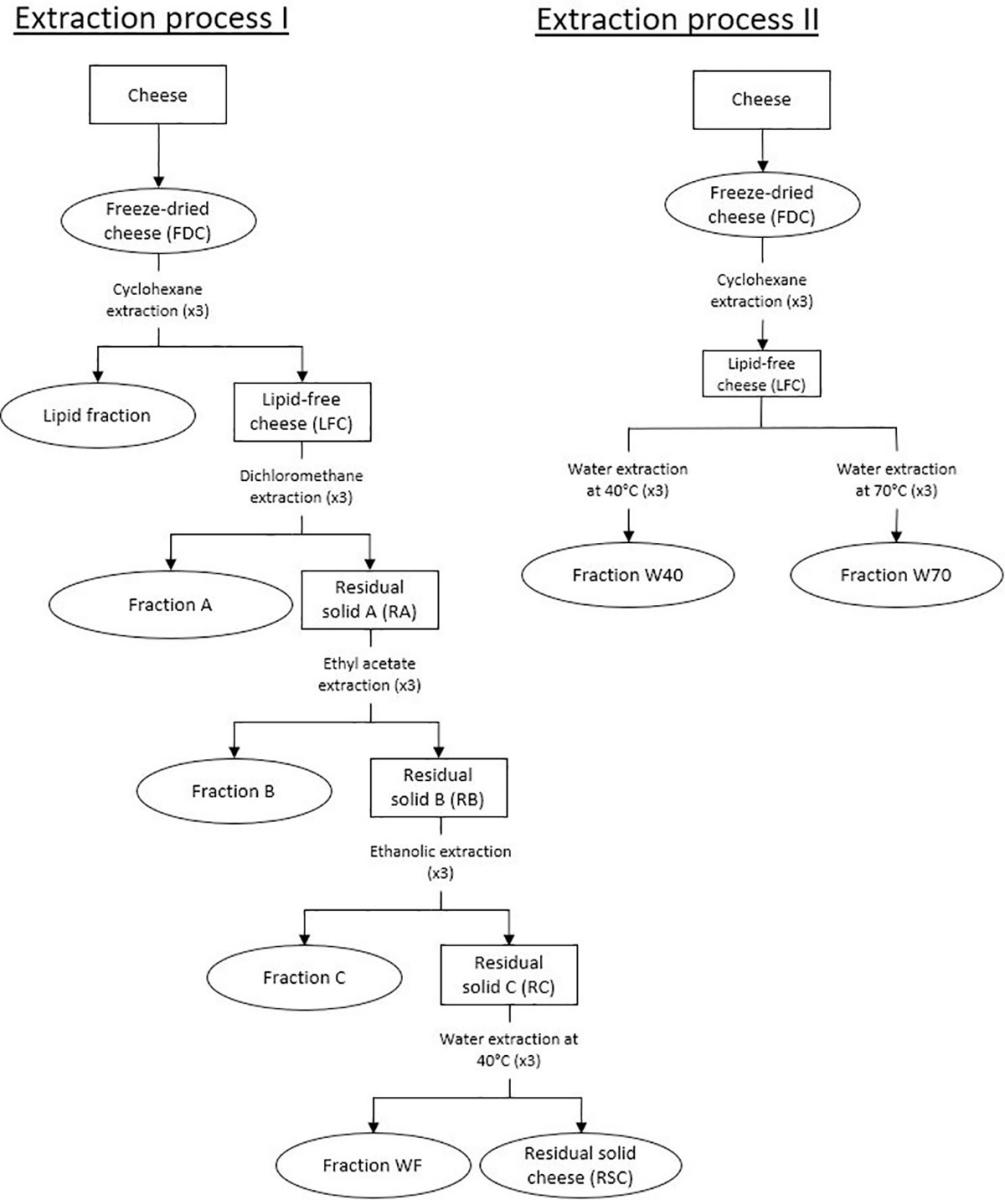
Preparation of cheese fractions. Rings indicate the cheese extracts used for biological tests (FDC, lipid fraction, fractions A, B, C, WF, W40, W70 and RSC).

Extractions were performed on the freeze-dried cheese, starting with cyclohexane (apolar solvent) used to extract lipid fraction. Then two different extraction processes were implemented to remove the metabolites. Extraction process I corresponded to fractional extraction. Extraction process II consisted in water extractions directly on the lipid-free cheese (LFC). The extraction procedures are resumed in the Fig 1.

#### Lipid extraction

A lipid extraction was performed to recover the apolar fraction of the cheese metabolites. The protocol was adapted from Bligh and Dyer (1959), Folch *et al*. (1957) and Manirakiza *et al*. (2001) [28–30]. Distilled cyclohexane (ratio 1/10 (w/w)) was added to FDC powder under mechanical agitation for 4 h. The resulting mixture was filtered with Büchner, concentrated under vacuum and the residue was dissolved in cyclohexane (ratio 1/10 (w/v)), then filtered again to eliminate the residue of the cheese and again concentrated under vacuum. The solvent extraction was performed three times under the same conditions at different times (4 h, 2 h and 1 h, respectively) to exhaust the cheese matrix. The resulting dry extracts were combined to obtain the final fraction, named lipid fraction.

#### Extraction process I

A fractional extraction was carried out on the LFC to collect most of the components from the cheese matrix. The procedure resulted in four successive solid/liquid extractions, increasing the polarity of the solvent at each step: dichloromethane, ethyl acetate, absolute ethanol and water.

Extractions were performed on the LFC with dichloromethane, followed with ethyl acetate and ethanol as shown in Fig 1. The protocol used was the same as for the lipid extraction for each solvent, but with a 1/10 (w/v) ratio. The final fractions were named fraction A, B and C, respectively.

An aqueous extraction was performed on the residual solid C (RC). The protocol was adapted from Polychroniadou *et al*. (1999) and Huma *et al*. (2018) [31, 32]. The residual solid C (RC) was mixed with HPLC grade water at a ratio of 1/10 (w/v) under mechanical agitation and held at 40°C for 1 h. After centrifugation (8000 rpm, 15 minutes; Avanti J26S XPI, Beckman Coulter, Brea, USA), the supernatant was concentrated under vacuum, filtered and evaporated before drying under vacuum. The extraction was performed three times under the same conditions to exhaust the cheese matrix. The resulting dry extracts were combined to obtain the final fraction, crushed with mortar and pestle and named fraction WF.

The residual solid cheese (RSC) was finely crushed with mortar and pestle and then by cryogenic grinding.

#### Extraction process II

A process was performed on the LFC, consisting in two extraction conditions with water at two different temperatures (40°C and 70°C) (Fig 1). LFC was mixed with HPLC grade water at a ratio of 1/10 (w/v) under mechanical agitation and held at 40°C (or 70°C) for 1 h. After centrifugation (8,000 rpm, 15 minutes; Avanti J26S XPI, Beckman Coulter, Brea, USA), the supernatant was concentrated under vacuum, filtered and evaporated before drying under vacuum. The extractions were performed three times under the same conditions, for each temperature, to exhaust the cheese matrix. The resulting dry extracts were combined to obtain the final fractions, crushed with mortar and pestle and named fraction W40 (extraction at 40°C) or W70 (extraction at 70°C).

Each fraction was stored at -25°C under argon in a waterproof container until use.

### Growth of *Escherichia coli* and heat-killed

*Escherichia coli* (*E*. *coli)* OP50 was used to feed the worms during the assay. The strain was provided by the *Caenorhabditis* Genetics Center (Minneapolis, MN, USA) and was grown on Lysogeny Broth medium at 37°C overnight. Microbial suspensions were pelleted for 15 minutes at 4,000 rpm (Rotofix 32A, Hettich Zentrifugen, Tuttlingen, Germany) and washed with M9 buffer (per L: 3 g of KH2PO4, 6 g of Na2HPO4, 5 g of NaCl, 1 mL of 1 M MgSO4) to a final concentration of 100 mg/mL. To obtain heat-killed (HK) *E*. *coli* (100 mg/mL), the resulting suspension was held 1h in a water bath at 75°C. The solution was kept at 4°C until use.

### Culture of *Caenorhabditis elegans*

The *Caenorhabditis elegans* N2 (wild-type) strain was provided by the *Caenorhabditis* Genetics Center (Minneapolis, MN, USA). The nematodes were grown and maintained at 20°C on Nematode Growth Medium (NGM) plates (per L: 3 g of NaCl; 2.5 g of peptone; 17 g of agar; 5 mg of cholesterol; 1 mM of CaCl_2_; 1 mM of MgSO_4_, 25 mL of 1 M potassium phosphate buffer at pH 6), supplemented with yeast extract (4 g/L) (NGMY) and seeded with live *E*. *coli* OP50 [33–35].

### *Caenorhabditis elegans* synchronization

The eggs and gravid worms were washed with M9 buffer and centrifuged for 2 minutes at 1,500 rpm. The pellet was resuspended in 5 mL of worm bleach (2.5 mL of M9 buffer, 1.5 mL of bleach, 1 mL of sodium hydroxide 5M) and vigorously shaken until adult worm body disruption. The action of the worm bleach was stopped by adding 40 mL of M9 buffer. The egg suspension was then centrifuged for 2 minutes at 1,500 rpm and washed twice with 20 mL of M9 buffer. The eggs were allowed to hatch under slow agitation at 25°C for 24 h in 20 mL of M9 buffer. The L1 larvae were settled by centrifugation at 1,500 rpm for 2 minutes and were resuspended in 1 mL of the remaining supernatant. One hundred microliters was then transferred onto NGMY plates, seeded with live *E*. *coli* OP50, and incubated until the worms reached the L4 / young adult stage [33–35].

### *Caenorhabditis elegans* lifespan assay

In order to determine the effect of cheese extracts on the worm longevity, a lifespan assay was performed with the *C*. *elegans* wild-type strain N2. An agar medium (per L: 3 g of NaCl and 6 g of agarose) was prepared and stored at 55°C. This medium was adapted from the NGM used for the culture of the *C*. *elegans* but was depleted as much as possible to avoid any fungal development during the assay. The medium was divided into aliquots and individually supplemented with cheese extracts at the appropriate concentration (Table 1), before being poured at 40°C into 24-well plate with 0.12 mM of FUdR. Each extract was introduced into the medium at 0.5% (w/v). Other concentrations were tested according to the nature of the extract, so as not to modify the physicochemical properties of the medium. To avoid any significant fungal development, which was commonly observed when using total cheese and lipid fraction, the medium was supplemented with amphotericin B for a final concentration of 1.6 μg/mL. The presence of the antifungal, or not, established two control conditions: with and without amphotericin B. A comparison was made between the lifespans of the worms in each of the two control conditions to determine whether the antifungal had an influence on nematode longevity. Amphotericin B was chosen because of its broad spectrum of action and because it does not have a toxic effect on *C*. *elegans* [36, 37]. After pouring, the wells were immediately transferred onto ice, to solidify the agar, and stored at 4°C until being use. The synchronous L4 worms were incubated on a supplemented agar medium (or agar medium for the control condition) with ~20 worms per well. They were fed with HK *E*. *coli* OP50 and kept at 20°C for the duration of the experiment. The wells were supplemented with food every 3 days to avoid starvation (20 μL of 100 mg/mL suspension). Day 0 corresponded to the first day when the worms were incubated on the medium. Live worms were scored each day until the death of all of the nematodes. A worm was scored as dead when it did not respond to a gentle mechanical stimulation. The total cheese and cyclohexane extract conditions were compared to the control condition with amphotericin B and food (CC2) whereas the other conditions were compared to the control condition with food only (CC1). This assay was performed as four independent experiments with three wells per condition and conducted side by side with the control conditions [38, 39].

**Table 1:** Concentration of the different cheese extracts used for supplementing the medium.

Fractions	Concentration (w/v)			
	0.1%	0.5%	1%	2%
**Freeze-dried cheese (FDC)**		X		
**Lipid fraction**	X	X		
**Fraction A**		X		
**Fraction B**		X		
**Fraction C**	X	X		
**Fraction WF (40°C)**		X	X	X
**Residual Solid Cheese (RSC)**		X		
**Fraction W40 (40°C)**		X	X	X
**Fraction W70 (70°C)**		X	X	X

Concentrations are expressed in percentage of extracts relative to the volume of medium.

### Statistical analysis

The data of the *C*. *elegans* survival assay were analysed using the Kaplan-Meier method, and differences were determined using the log-rank test with R software version 1.1.463. Differences were considered statistically significant if p-value ≤ 0.05. Conditions were compared to their respective control. Moreover, when it was possible, conditions at the same concentration were compared and the different concentrations of the same fraction were also compared.

## Results

### Development of the chemical extraction process

The goal was to develop a method of obtaining cheese fractions in order to investigate their biological capacities. As the composition of the cheese matrix was not fully described, we envisaged two extraction processes in order to extract most of compounds. Extraction process I consisted in successive extractions, increasing polarity solvents in order to remove the most compounds. Extraction process II consisted in removing the lipid fraction before performing two water extractions at two different temperatures (40°C and 70°C). For each solvent, extraction was performed three times to exhaust the cheese matrix and recover the most components. The Residual Solid Cheese (RSC) was also recovered at the end of the Extraction process I and represented 24.5% of the initial freeze-dried cheese (FDC) engaged. After each separation step, the yield of the dry extract was calculated. A comparison was made between the yields of each separation step for the same solvent in order to evaluate the efficiency of the different extractions (Table 2 for Extraction process I, Table 3 for Extraction process II). For most solvents, more than 60% of the final extract was recovered during separation step 1 (45.9% of the final yield of 47.9% in the case of the lipid fraction). These values were clearly higher than the yields of separation steps 2 and 3 involving the same solvent. For example, a yield of 45.9% was calculated for the lipid fraction in separation step 1, whereas yields for separation steps 2 and 3 were 1.9% and 0.1%, respectively. The significant differences in yields between the successive separation steps demonstrated the efficiency of the developed method to exhaust the cheese matrix and to recover the most compounds.

**Table 2:** Yields from Extraction process I in percentage (lipid fraction and fractions A, B, C and WF).

Fractions	Yields (%)			
	separation step 1	separation step 2	separation step 3	Final fraction
**Lipids**	45.9	1.9	0.1	47.9
**A**	1.3	0.2	0.1	1.6
**B**	0.08	0.03	0.01	0.12
**C**	0.9	0.5	0.4	1.8
**WF (40°C)**	13.1	4.9	3.0	21.0

**Table 3:** Yields from Extraction process II in percentage (Fractions W40 and W70).

Fractions	Yields (%)			
	separation step 1	separation step 2	separation step 3	Final fraction
**W40 (40°C)**	15.8	4.7	1.2	21.7
**W70 (70°C)**	16.2	2.4	0.5	19.0

### Effects of the antifungal on the nematode lifespan

**Fig 2:**
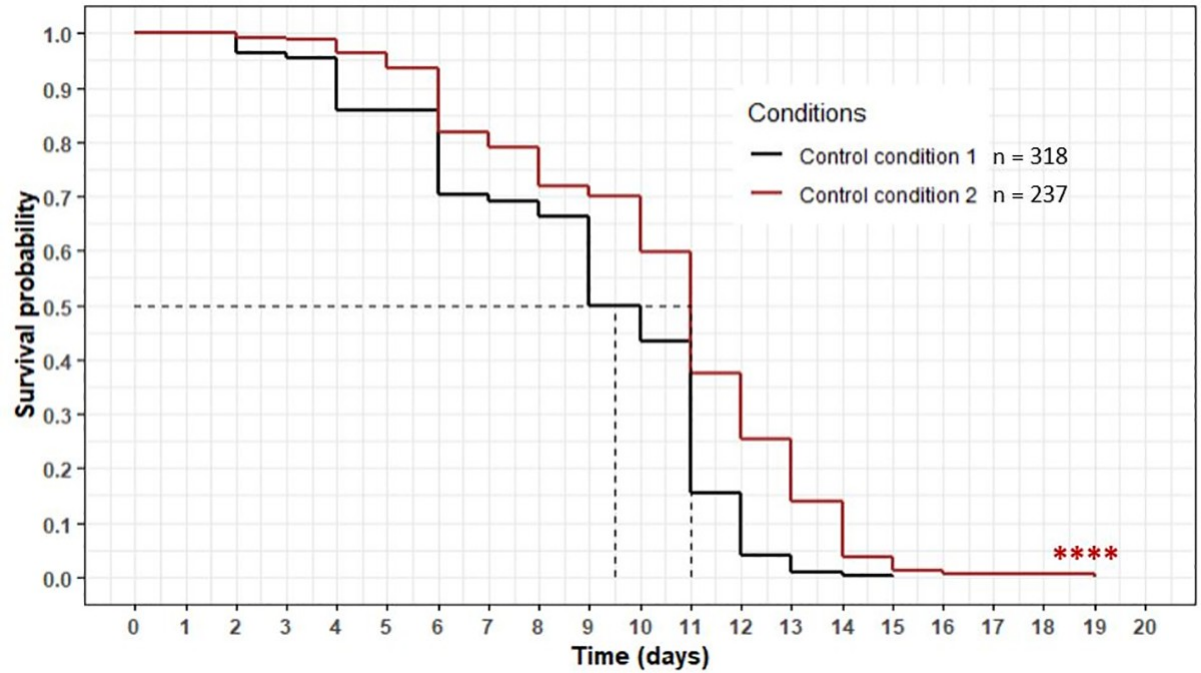
Influence of amphotericin B on *C*. *elegans* wild-type N2 strain lifespan. Comparison between CC1 and CC2. Worms were incubated on the medium (with or without antifungal) at day 0 and fed every three days with HK OP50. The asterisks indicate the *p-*values (log-rank test) (“*”: p <0.05; “**”: p <0.01; “***”: p <0.001; “****”: p <0.0001).

During the supplementation of the medium, an antifungal, amphotericin B, was added with the FDC and lipid fraction to avoid any significant fungal development that would hinder the ability to count the worms. A specific control condition with amphotericin B (CC2) was prepared to evaluate the effects of the antifungal on the nematode lifespan. To evaluate the effects of the antifungal, the relative position and the evolution of the survival curve of CC2 was compared with the control condition without antifungal CC1 curve (Fig 2).

We observed that the CC2 curve was significantly above CC1 curve (*p-*value <0.0001). The mean and maximum lifespan were significantly higher for CC2 (11 and 19 days, respectively) than for CC1 (9.5 and 15 days, respectively) (p <0.0001). These results demonstrated the impact of the antifungal on the nematode lifespan, justifying the use of a control with antifungal to evaluate the effect of the FDC and lipid fractions on longevity.

### Cheese extracts influenced the lifespan of *C*. *elegans*

**Fig 3:**
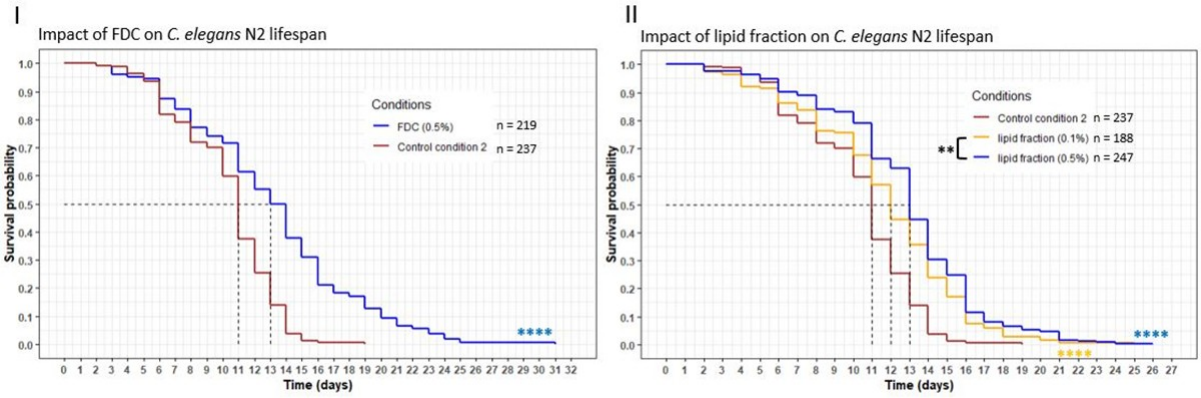
Influence of FDC and lipid fractions on *C*. *elegans* wild-type N2 strain lifespan. Worms were incubated on fractions at day 0 and fed every three days with HK OP50. The asterisks indicate the *p-*values (log-rank test) (“*”: p <0.05; “**”: p <0.01; “***”: p <0.001; “****”: p <0.0001). The asterisks around the curves are the comparisons with the control conditions. The asterisks around the legend are the comparisons between the fractions.

**Fig 4:**
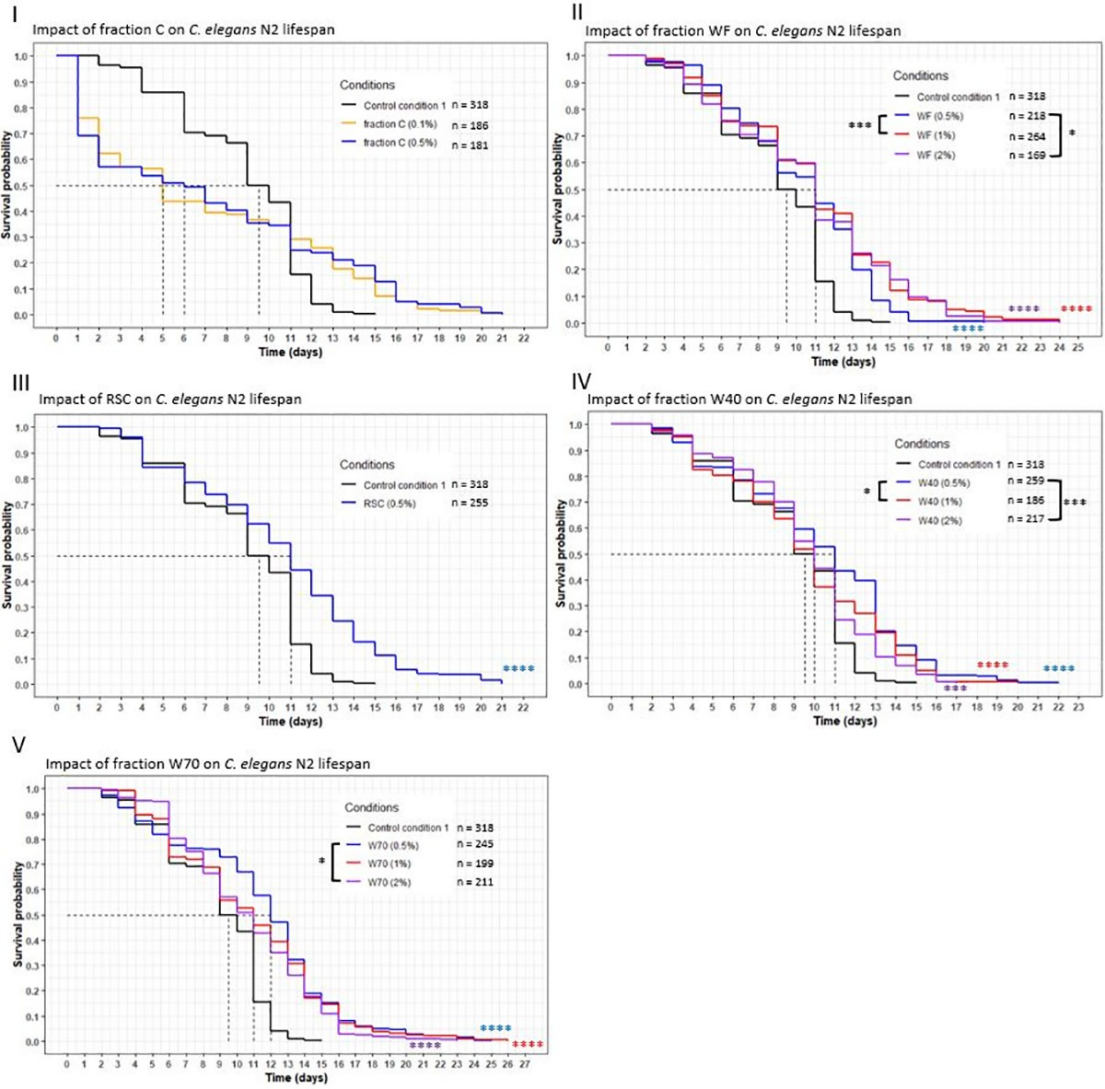
Influence of fractions E, WF, W40, W70 and RSC on *C*. *elegans* wild-type N2 strain lifespan. Worms were incubated on fractions at day 0 and fed every three days with OP50 HK. The asterisks indicate the *p-*values (log-rank test) (“*”: p <0.05; “**”: p <0.01; “***”: p <0.001; “****”: p <0.0001). The asterisks around the curves are the comparisons with the control conditions. The asterisks around the legend are the comparisons between the fractions.

The goal was to investigate the effect of cheese fractions on *C*. *elegans* longevity with a lifespan assay (Figs 3 and 4). Complementary information was taken into account when evaluating the effects of cheese fractions and whole cheese: a comparison between the relative position of the curves and their evolution, the mean and maximum lifespan and the percentage of the population still alive on cheese conditions when the worms of the control condition were all dead (Tables 4 and 5).

**Table 4:** Survival of *C*. *elegans* fed with FDC and lipid fractions and CC2 as a control.

Tested conditions	Concentration (w/v) (%)	Mean lifespan (days)	Maximum longevity (days)	Percentage of population alive at 19 days (%)	p-value
**Freeze-dried cheese (FDC)**	0.5	13	31	13	< 0.0001
**Lipid fraction**	0.1	12	25	3	< 0.0001
	0.5	13	26	5	< 0.0001
**CC2**	-	11	19	0	-

Mean lifespan, maximum longevity and the percentage of population alive are from survival curves. *p-*values were calculated by comparing conditions with CC2 using the log-rank test.

**Table 5:** Survival of *C*. *elegans* fed with fractions C, WF, W40, W70 and RSC and CC1 as a control.

Tested conditions	Concentration (w/v) (%)	Mean lifespan (days)	Maximum longevity (days)	Percentage of population alive at 15 days (%)	p-value
**Fraction C**	0.1	5	20	8	0.4
	0.5	6	21	13	0.5
**Fraction WF (40°C)**	0.5	11	20	5	< 0.0001
	1	11	24	12	< 0.0001
	2	11	24	16	< 0.0001
**Residual Solid Cheese (RSC)**	0.5	11	21	11	< 0.0001
**Fraction W40 (40°C)**	0.5	11	22	10	< 0.0001
	1	10	20	5	< 0.0001
	2	10	17	4	0.0004
**Fraction W70 (70°C)**	0.5	12	25	15	< 0.0001
	1	11	26	14	< 0.0001
	2	11	23	11	< 0.0001
**CC1**	-	9.5	15	0	-

Mean lifespan, maximum longevity and the percentage of population are from survival curves. *p-*values were calculated by comparing conditions with CC1 using the log-rank test.

Incubating worms with freeze-dried cheese (FDC) resulted in a significant increase in the longevity compared to the control condition CC2 (*p*-value <0.0001, Fig 3I), with the FDC curve above the CC2 curve all along the assay and a mean lifespan changing from 11 to 13 days (+ 18%). Another important observation was that 13% of the worm population on FDC supplemented medium were still alive at 19 days (maximum lifespan of the worms of CC2) and stayed alive until 31 days (+ 63%) (Table 4). The same observations were made with the worms incubated with the lipid fraction (Fig 3II). Curves of the lipid fraction were above the CC2 curve all along the assay with a significant difference (*p-*values <0.0001 for both concentrations). The mean lifespan increased from 11 to 12 days (+ 9%) (0.1% w/v) or 11 to 13 days (+ 18%) (0.5% w/v). A small proportion of the population on the medium supplemented with the lipid fraction was still alive at 19 days (5% maximum) and stayed alive until 26 days (+ 37%). A dose-response was also observed for the lipid fraction with the curve for 0.5% concentration significantly above the curve of 0.1% concentration (*p* = 0.006). FDC and lipid fraction showed a beneficial effect on the nematode lifespan. The difference with CC2 was significant, meaning the effect was not due to the antifungal added to the medium.

Fractions A and B killed the worms immediately after incubating them on the supplemented medium. Consequently, no data could be presented for these two conditions.

The survival of the worms incubated on fraction C presented a two-phase variation during the assay. In the first days, an important decrease in the population was observed (Fig 4I), with curves below the CC1 curve for both concentrations. This resulted in a mean lifespan lower than CC1 by 47% (at 0.1%) and 37% (at 0.5%) (Table 5). However, a few days after this initial decrease, an increase in the lifespan, to a maximum of 21 days, became apparent, and up to 13% of the population was still alive whereas the control condition died at 15 days. After killing half of the population within the first few days, the fraction C induced a beneficial effect from then on.

Incubating worms on fraction WF, W40, W70 or Residual Solid Cheese (RSC) also resulted in an extended longevity. Each concentration of these fractions significantly increased the mean lifespan compared to CC1 (*p*-value <0.0001) with the curves of the fractions above the CC1 curve (Fig 4II–4V). The mean lifespan increased between 5% and 26%, with the most important variation observed for W70 at 1% w/v (Table 5): the mean lifespan changed from 9.5 to 12 days. Longevity rose in all conditions, with a minimum increase of 13%, to a maximum increase of 73%, with W70 at 1% showing the best variation, going from 15 (CC1) to 26 days. Moreover, the worm populations incubated on these fractions were all still alive at 15 days, with up to 16% alive for WF at a concentration of 2%. For fractions WF, W40 and W70 a dose-response was observed. In general, there was a significant difference between the W40 curve at 0.5% and the curves of the other concentrations (*p* = 0.02 with 1%, *p* = 0.0004 with 2%), whereas for the same concentration of WF, the curve was significantly below the others (*p* = 0.003 with 1%, *p* = 0.02 with 2%). A significant difference was only observed for W70 between 0.5% and 2%, with the 0.5% concentration curve above the 2% concentration curve (*p* = 0.01). In sum, the beneficial impact of these fractions was shown by the increase in the longevity of the worms compared to that of the CC1 worms.

### Impact of the extraction conditions of the fractions prepared with water on worm lifespan

**Fig 5:**
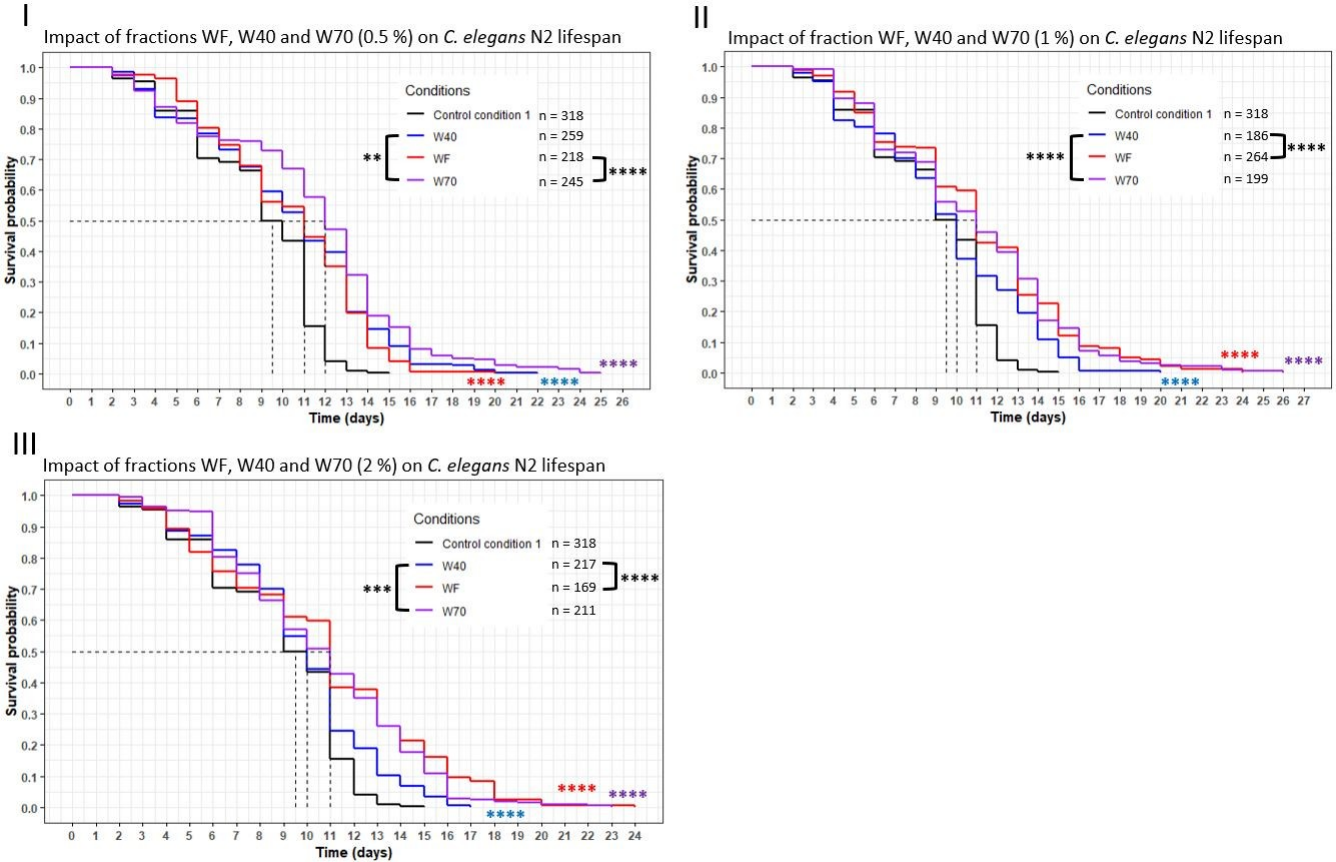
Comparison of the effect of aqueous fractions WF, W40 and W70 on *C*. *elegans* wild-type N2 strain lifespan. Worms were incubated on aqueous fractions at day 0 and fed every three days with OP50 HK. The asterisks indicate the *p-*values (log-rank test) (“*”: p <0.05; “**”: p <0.01; “***”: p <0.001; “****”: p <0.0001). The asterisks around the curves are the comparisons with the control conditions. The asterisks around the legend are the comparisons between the fractions.

Three different fractions were obtained with water extraction: WF from the Extraction process I, W40 and W70 from the Extraction process II. The worms were exposed to different concentrations (0.5, 1 and 2%) (Fig 5) of these fractions. At the 0.5% concentration, W70 showed the highest effect, with the curve significantly above the WF and W40 ones (*p* = 0.002 with W40; *p* <0.0001 with WF). The mean lifespan and maximum longevity were the highest for the fraction W70 at this concentration (Table 5). At the 1% and 2% concentrations, the survival curve of W40 was significantly below the WF (*p* = 0.0009 at 1%; *p* = 0.0004 at 2%) and the W70 (*p* <0.0001 for both concentration) curves. In both concentrations, the increase in mean lifespan and maximum longevity was lower for W40 (+5% and up to +33%, respectively) than for the other fractions (+16% and at least +53%, respectively). The nematodes did not show the same biological response to the fraction WF, W40 and W70, suggesting a variation in their composition.

## Discussion

### Determination of the optimal parameters to perform the longevity assay on the worms

To investigate the effects of cheese extracts on the nematode lifespan, we had to develop a method of bringing the worms into contact with them. Recent studies have demonstrated the possibility of providing the worms with plant extracts through two different methods. The first method consists in adding the extract to a liquid medium either in its solid form or dissolved in a solvent [26, 27, 40]. In some case, solvents such as Dimethyl sulfoxide (DMSO), were added to the medium to dissolve the plant extract. However, their possible toxic effects on the nematode are a concern, as reported by Wang *et al*. (2010) and Katiki *et al*. (2011) [27, 41], who observed a dose-effect reduction in longevity. A second method has been developed to supply the plant extracts by means of a less toxic solvent. Fan *et al*. (2011) and Wiegant *et al*. (2009) [38, 39] supplemented the NGM agar plates directly before incubating the worms. We adapted this second supplementation method to supply the worms with the cheese extracts in a homogeneous way. Preliminary trials were done with the NGM used for the culture of *C*. *elegans*. However, supplementing this medium with freeze-dried Cheese (FDC) and/or fractions resulted in a significant fungal development due to the microorganisms found in cheese and, thus, in some of the extracts, such as the lipid fraction. These microorganisms were not killed during the chemical extraction and proliferated in the medium during the lifespan assay, preventing us from visualising and determining the number of live worms. To solve this problem, the initial medium was depleted as much as possible. The cheese extracts were then added to the agar medium and homogenized before being poured in the wells. This modification limited the fungal development except in the FDC and the lipid fraction. In the case of these extracts, the microorganisms were still present and growing. To thwart this critical point, amphotericin B was added to avoid any significant fungal development. Breger *et al*. (2007) [36] and Huang *et al*. (2014) [37] demonstrated that this antifungal did not have a toxic effect on the nematode. However, this antifungal may have had a positive impact on the lifespan of *C*. *elegans*. Therefore, two control conditions were studied and compared, CC1 (without antifungal) and CC2 (with antifungal). The assay demonstrated a significant difference between the survival curves as shown in Fig 2. The antifungal tended to increase the longevity of *C*. *elegans* significantly, suggesting another source of nutrients through carbon intake via the amphotericin B. Consequently, to take into account the presence of the antifungal in the medium, the FDC and the lipid fraction were compared with the CC2.

Another parameter had to be considered to evaluate the effects of the extracts on the longevity. Indeed, the age of the worms, when incubated on the medium, has a notable impact on the results. So, to avoid this age effect, longevity studies were performed with L4 synchronized worms. Once the worms were incubated on supplemented medium, food was placed on the medium. The development of this method allowed for an efficient measure of the effects of the cheese extracts on the nematode lifespan.

In our experiment, the worms were fed their usual diet of *E*. *coli* OP50 because this customary food could not be replaced by the cheese extracts. To avoid any potential interaction between the bacterial food of the worms, *E*. *coli* OP50, and the cheese extracts, the bacteria were heat-killed before being given to the worms. This was done to ensure that the live bacteria could not consume the cheese metabolites or produce compounds in response to their presence, changing the biological effect of the cheese extracts. Killing the bacteria did not have any influence on the lifespan of the worms compared to the live bacteria, as demonstrated by Couillault and Ewbank (2002) [42]. This strategy, to use heat killed OP50, allowed for an efficient measure of the biological effects of the fractions on the longevity.

### Beneficial effects of cheese fractions on *C*. *elegans* lifespan

Our results demonstrated that the extracts exhibited different impacts on worm longevity. The fractions A and B contained compounds that had a negative impact on *C*. *elegans*. The fractions instantly killed the worms upon incubating them on the medium.

The fraction C, obtained with ethanol, engendered a two-phase reaction during the longevity assay. During the first days of the assay the fraction killed most of worms. However, after three days, it appeared to reinforce the surviving worms by extending their longevity. As the solvent was evaporated, these results allowed two hypotheses to be put forward: the fraction must have contained toxic compounds in low concentration which harmed and killed the worms. Once the concentration decreased, the beneficial effects of the metabolites took over and increased the longevity of the surviving worms. The second hypothesis was that toxic compounds were not necessarily in low concentration, but the worms adapted, resulting in an improved longevity after a few days. As this drastic decrease in the survival curve at the beginning of the assay was not observed for the other fractions, the compounds with a negative impact might be specific to this fraction and, consequently, could be extracted with ethanol alone.

All of the other fractions demonstrated a beneficial effect on the nematode lifespan. Concerning the freeze-dried cheese (FDC) and the lipid fraction, the comparisons were carried out with the CC2, to take into account the presence of the antifungal in the medium. As they significantly increased the lifespan of the worms compared to the CC2 (*p* <0.0001 for both fractions), this observation showed that the beneficial effects cannot be attributed to the antifungal alone, but to the presence of the extracts themselves. The fractions WF, W40, W70 and Residual Solid Cheese (RSC) presented an increased lifespan compared to the CC1 (*p* = 0.0004 for W40 at 2%, *p* <0.0001 for other conditions). In some cases, a dose-response could be observed, with differences between the concentrations for WF, W40 and W70. The different effects observed suggest that they could be correlated with the composition of the fractions. Further chemical analyses and genetic studies are necessary to investigate the nature of the compounds and the signalling pathways involved in the differences on *C*. *elegans* lifespan.

All these results validated, as evidenced by the variations of the biological responses of the worms, that the medium supplementation was efficient with every fraction. This study showed that *C*. *elegans* was a pertinent model for screening the effects of cheese extracts on the longevity. In fact, most fractions were able to increase the longevity significantly in comparison with their respective control condition, with a dose-response in some cases. The next step in this methodology would be to study the mechanism of action of the fractions by using nematodes mutants. Some signalling pathways have already been described to be involved in the mechanism of the longevity [35, 40] and could represent a new study target.

### Comparison of the effects of extracts WF, W40 and W70 on nematode longevity

As discussed above, the longevity assay demonstrated the beneficial effects of the extracts WF, W40 and W70 on the worms. Each extract increased the longevity of the worms significantly compared to the CC1. Another result given by the assay was the difference in their biological effect when introduced into the same concentration. At 1% and 2%, the extract W40 presented the lowest effect, as its curve was significantly below the two other extracts. Concerning the 0.5% concentration, W70 showed the highest effect compared to the other two extracts. The extracts prepared with water exhibited a beneficial effect on worm longevity. The variations in the responses of the worms may be correlated with the variations induced by the extraction conditions, in the composition of the extracts. Further chemical analyses are needed to investigate and validate this hypothesis. All these results demonstrated that the Extraction process II was an efficient process to obtain extracts with a beneficial effect on longevity. Indeed, the extracts were easier to obtain using 2-step solvent extractions in comparison with the Extraction process I. Moreover, the Extraction process II was performed using water, essentially, which is a most suitable solvent for industrial development and applications of using cheese fractions for their health benefits.

Comparing the biological effects of different extracts prepared with the same solvent was made possible thanks to *C*. *elegans*, thereby classifying this nematode as an efficient *in vivo* model. Thus, the methodology developed in this work will be used for a bioguided approach for the screening of the biological properties of different extracts.

## Conclusion

This study allowed the development of a methodology to isolate the metabolites from the cheese matrix and to evaluate the biological effect of the extracts using an *in vivo* test. This work validated the method of bringing the worms into contact with the cheese extracts. We demonstrated that fermented foods, especially raw-milk cheese, could promote a beneficial effect on the *C*. *elegans* longevity. The results confirmed the beneficial role of some cheese extracts (FDC, RSC, WF, W40, W70 and lipid fraction), in increasing the mean and maximum lifespan of the worms. In order to deepen their characterisation, the determination of the chemical composition of extracts and the identification of the biological mechanisms involved in their effects will be the objective of further investigations. The methodology developed in this study could be used for further biological studies or could be applied to other fermented foods.
